# Elevated CD36 expression correlates with increased visceral adipose tissue and predicts poor prognosis in ccRCC patients

**DOI:** 10.7150/jca.30989

**Published:** 2019-07-25

**Authors:** Wen-Hao Xu, Yuan-Yuan Qu, Jun Wang, Hong-Kai Wang, Fang-Ning Wan, Jian-Yuan Zhao, Hai-Liang Zhang, Ding-Wei Ye

**Affiliations:** 1 Department of Urology, Fudan University Shanghai Cancer Center, Shanghai 200032; 2 Department of Oncology, Shanghai Medical College, Fudan University, Shanghai 20032, P.R. China; 3 The Obstetrics & Gynecology Hospital of Fudan University, State Key Lab of Genetic Engineering, School of Life Sciences and Collaborative Innovation Center of Genetics & Development, Fudan University, Shanghai 200032, P.R. China

**Keywords:** *CD36*, visceral adipose tissue, subcutaneous adipose tissue, body mass index, clear cell renal cell carcinoma

## Abstract

***Objective*:** Growing evidence has proved obesity one of the confirmed important etiologic indicators for renal cell carcinoma (RCC). *CD36* is underpinned to be involved in adipose absorption, but its role in clear cell renal cell carcinoma (ccRCC) remains unclear. This study aimed to investigate the mRNA expression of *CD36* in anthropometric measures of adipose tissue and defining its value in predicting prognosis in ccRCC patients.

***Methods*:** Real-Time qPCR gene expression analysis was detected from 367 paired ccRCC and adjacent normal tissues. Distributions of categorical clinical-pathological data together with levels of *CD36* expression were compared with χ^2^-test in a contingency table. Subcutaneous adipose tissue (SAT) and visceral adipose tissue (VAT) were measured by magnetic resonance imaging (MRI) and identified at the level of the umbilicus. Pearson's correlation coefficient was utilized to quantify relations between body mass index (BMI), VAT%, SAT and *CD36* expression respectively. Partial likelihood test from univariate and multivariate Cox regression analysis were developed to address the influence of independent factors on progression-free survival (PFS) and overall survival (OS). The Kaplan-Meier method and log-rank test were performed to assess the survival benefits between discrete levels.

***Results*:** In the current study, *CD36* mRNA was demonstrated highly expressed in ccRCC compared with normal tissues. In addition, *CD36* mRNA expression was significantly increased in patients with advanced TNM stage (*p*=0.003, *p*<0.001, *p*<0.001), and high VAT% (*p*=0.004). Pearson's correlation coefficient indicated that *CD36* amplification positively correlated with BMI (*r*=0.117, *p*=0.025), VAT% (*r*=0.465, *p*<0.001), while negatively associated with SAT (*r*=-0.296, *p*=0.002). Median PFS was 60 months and OS was 99 months. Meanwhile, ccRCC patients with elevated *CD36* expression held shorter PFS and OS, with hazard ratios [HR; 95% confidence interval (CI)] of 4.873 (3.300-7.196, *p*<0.001) and 4.610 (2.956-7.189, *p*<0.001). In 104 cases with available MRI scans, VAT was significantly correlated with poor PFS and OS, with HR of 2.556 (1.036-6.310, *p*<0.042) and 3.291 (1.034-10.477, *p*<0.044). A total of 100 significant genes were obtained from GSEA, and *CD36* was found involved in the most significant pathways including fatty acid metabolism, UV response, angiogenesis and transforming growth factor beta (TGF-β) signaling pathways.

***Conclusion*:** In conclusion, our study first reveal that elevated *CD36* mRNA expression is positively correlated to distribution of abdominal adipose, particularly VAT%, which, in addition, notably predicts poor prognosis in ccRCC patients.

## Introduction

Renal cell carcinoma (RCC) has become one of the most common malignancy in the genitourinary system, and accounts approximately 3% of adult malignant tumors and 2% of all cancer deaths 1. The worldwide morbidity and mortality rates of RCC are growing approximately 2-3% per decade 2. Until the last few years, the rates are stabilizing or declining in many developed countries 3. Clear cell renal cell carcinoma (ccRCC) is the most predominant histological subtype of RCC, responsible for about 75% of cases. The incidence of the disease varies among demographic, geographic, and to a lesser extent, hereditary factors. Nowadays, many prevalence of factors contributing to RCC have been identified, especially obesity and physical inactivity.

Cluster of differentiation 36 (*CD36*) is encoded by the *CD36* gene. As an integral membrane protein present on the surface of various cell types in vertebrate 4, *CD36* antigen was involved in adipose metabolism and individual obesity 4-7. For example, Pepino et al. detected genetic bases for different expression of the *CD36* receptor in obese individual, demonstrating significant obesity sensitivity in a significant relationship with expression of* CD36* receptor 7. In 2017, Pascual et al. found *CD36* participated in fatty acid intake on the membrane of metastatic tumor cells, including melanoma, breast, bladder, lung and ovarian carcinoma. Interestingly, tumor cells completely stopped metastasis in mouse models when *CD36* expression was knockdown 8. Therefore, *CD36* may affect the tumorigenesis of ccRCC through underlying lipid metabolism variations.

Several studies demonstrated that body mass index (BMI) was one of the relative risk factors in ccRCC patients 9-12. Also, it is noteworthy that BMI is globally accepted as a valid and convenient anthropometric measurement of obesity classifications. Recently, many studies have controversies on the accuracy of BMI for individual body fat assessment and metabolic risk 13-15. So, it is necessary to introduce new measurements to indicate different body adipose depots, and their potential value in pathogenesis of ccRCC 16, 17. In 2010, Ibrahim MM indicated that the anatomy and biological functions distinguished greatly between subcutaneous adipose tissue (SAT) and visceral adipose tissue (VAT) 18. Different from SAT, VAT is surrounded between organs in abdominal cavity, and was deemed to predispose individual to type II diabetes, inflammation, and other obesity-related diseases 19. The relationship between VAT and RCC was also reported in recent years. One retrospective study in China found that increased VAT was significantly associated with high Fuhrman grade in patients with clinical T1a RCC 20. The visceral obesity percentage (VAT%), calculated using the formula VAT%=[(AP-SAT)/AP]×100%, was deemed significant in clinical research and especially associated with disease recurrence for localized RCC patients 21. The increasing insights on fat distribution provide promising pathways to predict the occurrence of ccRCC and to further prevent from this malignancy.

To investigate the *CD36* mRNA expression in anthropometric measures of abdominal adipose tissue and defining its value in ccRCC patients, we enrolled 367 patients who have received previous radical nephrectomy in our institution. We hypothesized that *CD36* mRNA expression correlated with VAT%, and predicted aggressive progression and poor prognosis in ccRCC patients.

## Materials and methods

### Patients and Variables

A total of 367 ccRCC patients, who have underwent radical nephrectomy in the Department of Urology of Fudan University Shanghai Cancer Center (FUSCC) (Shanghai, China) from Aug 2008 to Sept 2017, were consecutively recruited in analyses, with electronic medical records or pathology reports available. Clinical and pathological parameters, specifically age at surgery, BMI, clinical manifestation, tumor laterality, tumor size, TNM stage, International Society of Urological Pathology (ISUP) grading classification and anthropometric measures of obesity on magnetic resonance imaging (MRI) (n=104), were summarized in Table 1. Tissue samples, including ccRCC and normal tissues, were collected during surgery and available from FUSCC tissue bank. All of the study designs and test procedures were performed in accordance with the Helsinki Declaration II. The Ethics approval and consent to participate of the current study was approved and consented by the ethics committee of FUSCC.

### Anthropometric measures of obesity

**Figure 1:**
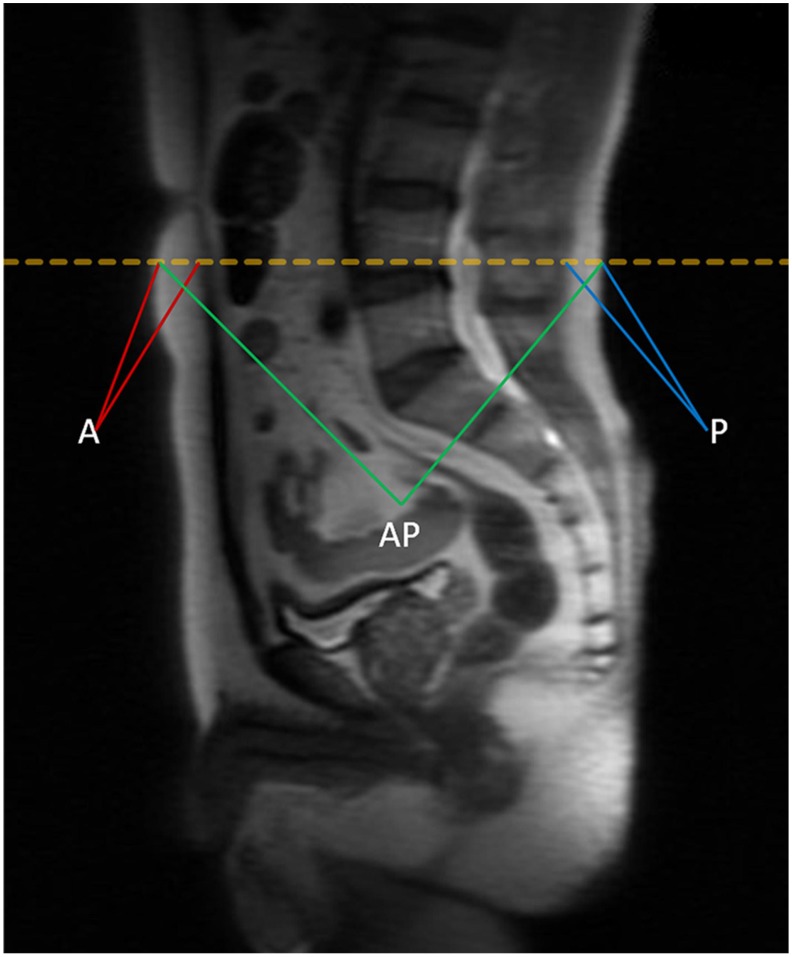
The quantity of anthropometric measures of SAT area was measured by MRI with T2-weighted sagittal localization images. All images were identified at the level of the umbilicus (approximately L4-L5 marked in orange dotted line) for 367 patients in supine position. A in maroon or P in blue represents measurement of the anterior or posterior abdominal adipose thickness respectively, and anteroposterior diameter (AP) was measured in green lines. The value of SAT was calculated as the sum of anterior abdominal fat and posterior abdominal fat (SAT=A+P). The visceral obesity percentage was defined as VAT%, which was calculated using the formula VAT%=[(AP-SAT)/AP]×100%.

BMI is defined as the weight (kg) divided by the square of body height (m), generally expressed in units of kg/m^2^. The quantity of anthropometric measures of SAT area was measured by MRI with T2-weighted sagittal localization images. All images were identified at the level of the umbilicus (approximately L4-L5 marked in orange dotted line) for 367 patients in supine position in Figure 1, and then averaged for the final measurement. A in maroon or P in blue represents measurement of the anterior or posterior abdominal adipose thickness respectively, and anteroposterior diameter (AP) was measured in green lines 22. Each MRI was reviewed by 2 experienced radiologists independently to determine the accuracy of measurement. The value of SAT was calculated as the sum of anterior abdominal fat and posterior abdominal fat (SAT=A+P). The visceral obesity percentage was defined as VAT%, which was calculated using the formula VAT%=[(AP-SAT)/AP]×100%.

### Real-Time Quantitative PCR analysis

Total RNA sequence was extracted using TRIzol reagent (Invitrogen, Carlsbad, CA) from 367 paired tumor and para-carcinoma normal samples. Total RNA reverse-transcribed reaction was performed using the SuperScript First-Strand cDNA Synthesis System (Invitrogen, Carlsbad, CA). ABI Prism 7900 Sequence Detector (Applied Biosystems) was utilized to realize Real-time PCR reactions. Forward PCR primers was 5'- GGCTGTGACCGGAACTGTG-3' and reverse primers was 5'- AGGTCTCCAACTGGCATTAGAA-3'. According to SYBR Green PCR master mix (Applied Biosystems) manufacturer protocols, a total of 10 μL reaction mixture was prepared for each test. Specific PCR operating cycles conditions for *CD36* and β-action were performed as follows: denaturation at 95℃ for 3 min, followed by 45 cycles of denaturation at 95℃ for 20 sec, annealing at 60℃ for 20 sec, extension at 68℃ for 20 sec, and measurement at 80℃ for 20 sec, followed by a final extension at 72℃ for 5 min. The *CD36* mRNA expression was represented as ΔCt = Ct_(CD36)_ - ΔCt_(β-actin)_. Relative expression in ccRCC was represented using the ratio of *CD36* expression in Tumor/Normal tissues (T/N). “Few *CD36* expression”, “Low *CD36* expression”, “Middle *CD36* expression” and “High *CD36* expression” denote the T/N ratio of *CD36* mRNA expression of less than 1, more than 1 and less than 5, more than 5 and less than 10, and more than 10, respectively. X-tile software was utilized to take the cut-off value: point of 5 and 10, in concordance of which overall participants were divided to three groups: *CD36* expression high, middle and low.

### Statistical analysis

To figure out the associations of different *CD36* mRNA expression sets with clinicopathological characteristics, χ^2^-test was performed to compare the distribution of categorical data between groups. Pearson's correlation coefficient was utilized to quantify relations between BMI, VAT%, SAT and levels of *CD36* expression. The primary end point was OS for patients who are alive for a certain period of time, which was evaluated from the date of radical nephrectomy to the date of death or last follow-up. Progression-free survival (PFS), as the secondary end point, was the length of time from the initiation of surgery until the date of progression or the start date of a second-line treatment or the date of death, whichever occurred first. The follow-up duration was estimated using the Kaplan-Meier method with 95%CI and log-rank test in separate curves. Univariate and multivariate analysis were performed with Cox logistic regression models to find independent predictors. Cox regressions on 104 participants enrolled with available MRI scan were independently analyzed to evaluated confounding covariates including BMI, A, P, VAT%, SAT on survival as supplements. Statistics analyses were performed with SPSS software (version 23.0, Inc, Chicago, IL, USA). All hypothetical tests were two-sided and *p*-values less than 0.05 were considered significant in all tests.

### Data processing of GSEA

Datasets from the Cancer Genome Atlas database were implemented with GSEA method using the Category version 2.10.1 package. For each separate analysis, Student's-t-test statistical score was performed in consistent pathways and the mean of the differential expression genes was calculated. A permutation test with 1000 times was used to identify the significantly changed pathways. The adjusted P values (adj. P) using Benjamini and Hochberg (BH) false discovery rate (FDR) method by default were applied to correct for the occurrence of false positive results 23. The significant related genes were defined with an adj. P less than 0.01 and FDR less than 0.25.

## Results

In this study, research was conducted in three series. In the first series, progression and prognostic value of *CD36* mRNA expression in ccRCC patients were assessed; in the second series, relationships between *CD36* expression levels and anthropometric measures of obesity tissue were illustrated; in the third series, the role of VAT% predicts survival benefits in ccRCC patients.

### Clinicopathological characteristics of the cohorts

As shown in Table 1, patients with increased *CD36* mRNA expression significantly correlated with advanced T (*p*=0.003), N (*p*<0.001), M stage (*p*<0.001), and higher VAT% (*p*=0.004). Chi-square test showed that baseline data were balanced on the distribution of categorical data, including age at surgery, sex, BMI, clinical manifestation, laterality, ISUP grade and SAT (*p*>0.05).

### Expression of CD36 in FUSCC

**Figure 2:**
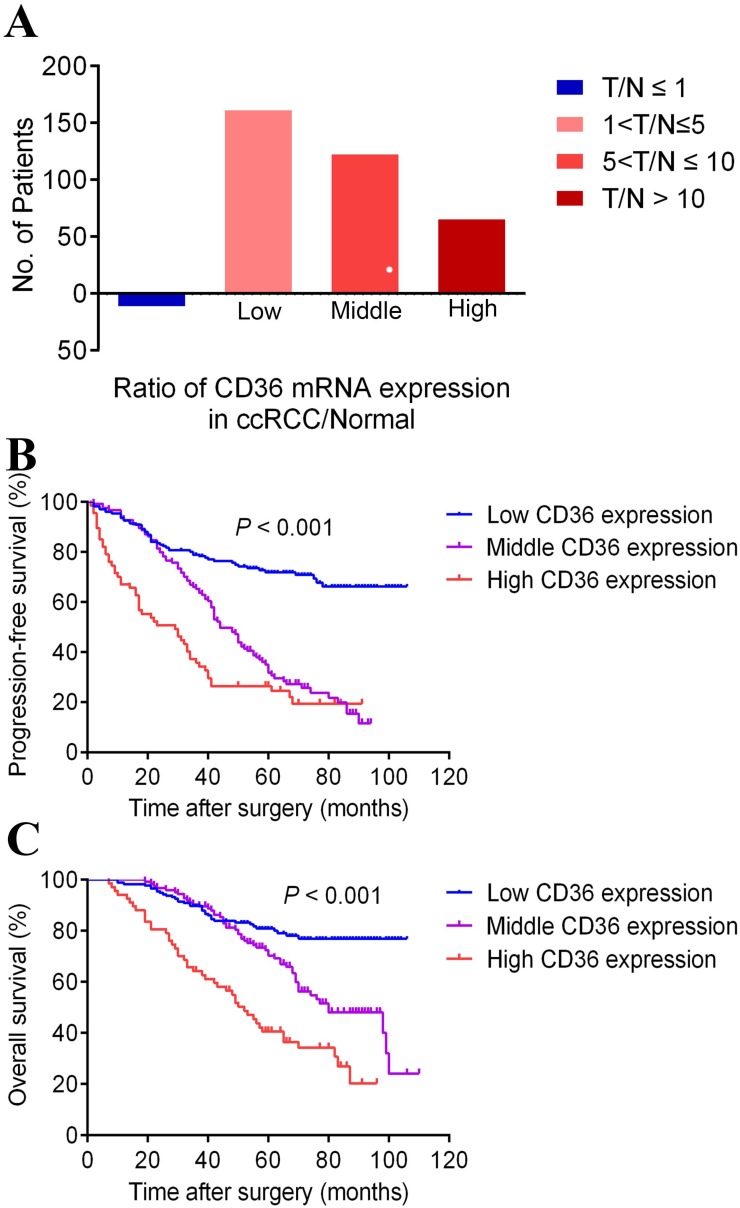
The percentage of different *CD36* mRNA expression in patients with different T/N, which was define as the ratio of *CD36* expression in ccRCC/Normal tissue in panel A. Kaplan-Meier survival analyses on different *CD36* expression groups with PFS (B) and OS (C) in the included 367 ccRCC patients. Compared with middle and low *CD36* expression, high *CD36* expression is significantly correlated with poor PFS (*p*<0.01) and OS (*p*<0.01).

To analyze the *CD36* mRNA expression profile of ccRCC tumor tissue, Real-time PCR revealed participates distribution concerning the ratio of T/N was dramatically different (3.5% in few *CD36* expression, 44.4% in low *CD36* expression, 33.8% in middle *CD36* expression and 18.3% in high *CD36* expression) provided in Figure 2A.

### Cox regression analyses and survival outcomes of the cohorts

In univariate and multivariate models, traditional prognostic factors, specifically T, N, and M stage, were still relevant to PFS and OS in ccRCC patients, indicating a fine representativeness of the population in the cohort of current research. Importantly, subgroups of *CD36* expression (Low vs. middle) and *CD36* expression (low vs. high) showed that *CD36* amplification markedly associated with poor PFS (*p*<0.001) and OS (*p*<0.001) for ccRCC patients both in Cox logistic regression analysis. BMI, with 25kg/m^2^ cutoff, was only significant in PFS (*p*=0.029) in univariate model of all Cox regression analyses. Besides, age at surgery and ISUP grade (1-2 vs. 3-4) were significant both in PFS (age: *p*=0.014, ISUP grade: *p*<0.001) and OS (age: *p*=0.012, ISUP grade: *p*<0.001) merely in univariate Cox regression. The other factors, including sex, clinical manifestation, tumor laterality and tumor size, were not assessed as prognostic indicators of PFS and OS in our study (*p*>0.05) (Table 2 and Table 3).

Survival curves suggested that patients with elevated *CD36* mRNA levels showed poorer PFS and OS (*p*<0.001). What's more, median PFS was 60 months and OS was 99 months in ccRCC patients. The median PFS in patients with middle and high *CD36* expression levels were 44 and 29 months, and median OS were 80 and 52 months, respectively. Data was not available in patients with low *CD36* expression due to their favorable prognosis, and median PFS and OS were not achieved by the time of last follow-up.

### VAT% is a better obesity indicator for ccRCC

**Figure 3:**
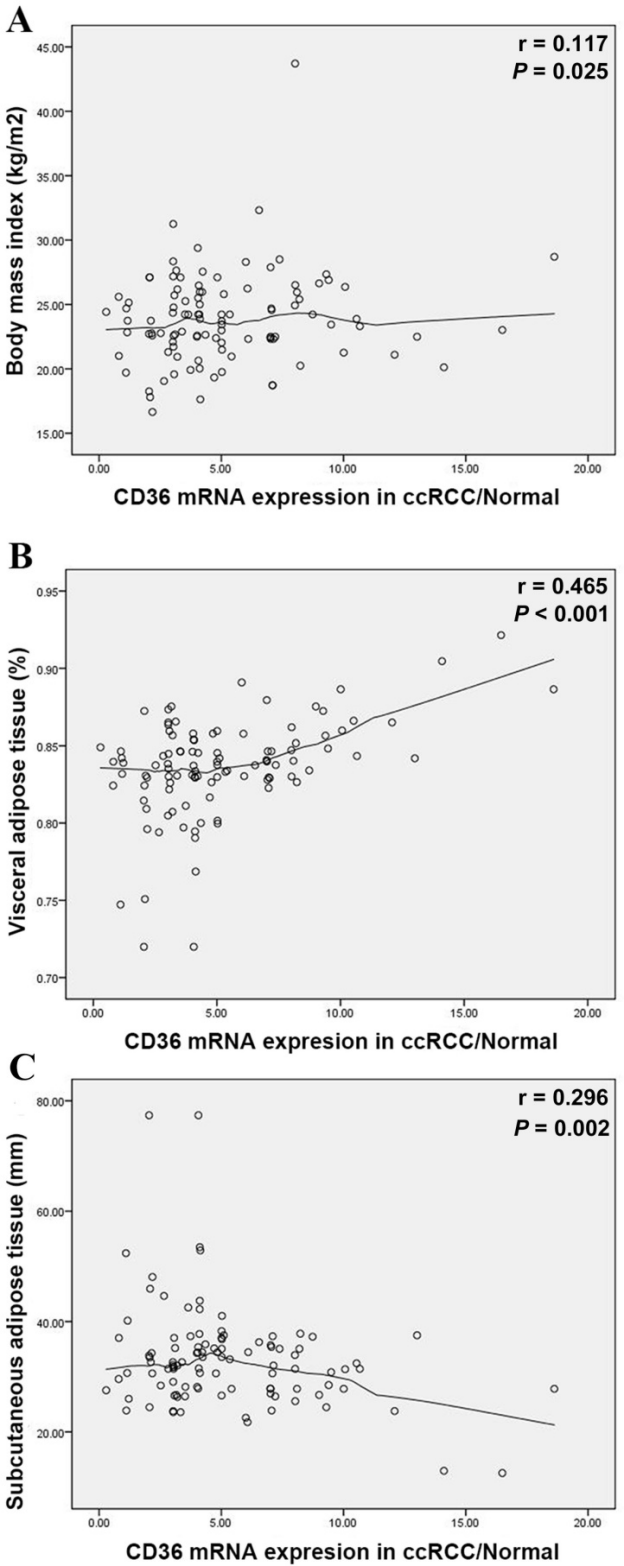
Scatterplots of the ratio of *CD36* mRNA expression in ccRCC/Normal tissues compared with BMI (A), VAT% (B) and SAT (C). Locally weighted scatterplot smoothing curves were fitted in plots. Pearson's correlation coefficient shows the T/N ratio was positively correlated with the BMI (*r*=0.117, *p*=0.025), VAT% (*r*=0.465, *p*<0.001) and negatively correlated with SAT (*r*=-0.296, *p*=0.002). VAT%: visceral obesity percentage; SAT: subcutaneous adipose tissue.

Elevated *CD36* mRNA expression correlated with higher VAT (%, *p*=0.004) in Table 1, yet insignificant with BMI (kg/m^2^, *p*=0.092). Meanwhile, *CD36* expression closely related to survival prognosis of ccRCC patients. To verify VAT% a more accurate predictor than BMI, we measured relations between abdominal adipose distribution and *CD36* mRNA expression. Pearson's correlation coefficient illustrated positive trend between BMI and T/N ratio (*r*=0.117, *p*=0.025) in Figure 3A. In parallel to BMI, T/N ratio was positively correlated with the VAT% (*r*=0.465, *p*<0.001) (Figure 3B), while negatively to SAT (*r*=-0.296, *p*=0.002) (Figure 3C).

Considering MRI scans were available only in 104 cases, in Supplementary Table 1 and Supplementary Table 2, multivariate Cox regression analyses of PFS and OS indicated that high VAT% was significantly related with poor PFS (HR=2.56, *p*=0.042) and OS (HR=3.291, *p*=0.044), while BMI was not independent covariate affecting survival.

### Significant genes and pathway obtained by GSEA

**Figure 4:**
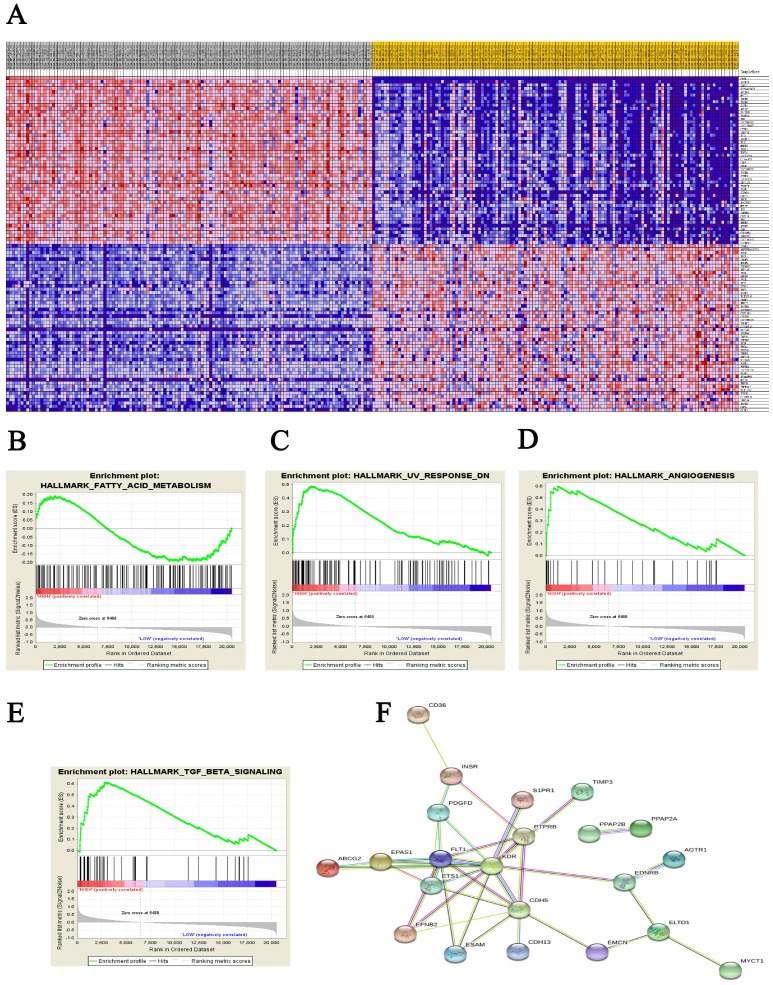
Datasets from public online database were implemented with GSEA method. For each separate analysis, Student's-t-test statistical score was performed in consistent pathways and the mean of the differential expression genes was calculated. A permutation test with 1000 times was used to identify the significantly changed pathways. The adjusted P values (adj. P) using Benjamini and Hochberg (BH) false discovery rate (FDR) method by default were applied to correct for the occurrence of false positive results. The significant related genes were defined with an adj. P less than 0.01 and a FDR less than 0.25.

A total of 100 significant genes were obtained from GSEA, and the genes with positive correlation were plotted. Besides fatty acid metabolism, *CD36* was found involved in the most significant pathways including UV response, angiogenesis and transforming growth factor beta (TGF-β) signaling pathways. The details were illustrated in Figure 4.

## Discussion

To clarify the effect of elevated *CD36* mRNA expression of ccRCC, we detected anthropometric measures of adipose distribution on MRI and observed that *CD36* mRNA amplification was significantly associated with increased VAT%. More importantly, patients with elevated *CD36* expression were exposed to poor PFS and OS after adjusting for all confounding covariates. It opens up a novel way for *CD36* mRNA expression to affect the pathogenesis of ccRCC by underlying adipose metabolism variation.

Recently, several studies in related fields deeply demonstrated the role of adipose and related genes in tumor incidence and prognosis. In January 2017, Lin H et al. found that fatty acid oxidation was vital energy source for the metabolism and proliferation of malignant glioma cells 24. It has upended long-held recognition on the primary energy source of tumor cells. Likewise, another research on Nature further suggested that, in addition to providing the “fuels”, fat may also “pave the way” for the spread of cancer cells in lymphangiogenesis 25. More importantly, a similar observation has been documented by Pascual et al., who revealed that *CD36* was responsible for the intake of fatty acid on the membrane of metastatic tumor cells, and metastasis was stopped after blockade of *CD36* in mouse models 8. Elevated *CD36* was underpinned associated with individual obesity 5, 26, 27, especially with VAT% in accord with previous studies emphasizing its clinical value 20, 28, 29. For example, it was proved that increased visceral-subcutaneous fat ratio was able to predict atherosclerosis 30, 31. Interestingly, an inverse association between specific type of carcinoma and atherosclerosis has been reported 32, which made a plausible explanation from another perspective to clarify the relationship of *CD36* and carcinogenesis.

Although BMI has been demonstrated risk indicator of adiposity related to neoplasia 9, 33, 34, as a traditional categorical variable, BMI may fail to adequately reflect the aggregate risk of ccRCC responses^35^. The role of BMI for individual body fat assessment was challenged for its rough estimation, including region, race diversity and failing to distinguish fat distribution 13-15. However, relative to BMI, less is known concerning relationship between body adipose depots and tumorigenesis. Emerging evidence indicated that hormonal and metabolic disorders related to excess adiposity are largely driven by VAT 36-38. Meanwhile, several investigations have documented and underpinned VAT as underlying mediator of adiposity triggering carcinogenesis, such as in colorectal adenomas 39, esophageal adenocarcinoma 40 and prostate cancer 22, 41. Our previous studies also suggested VAT correlated with high Fuhrman grade and pathological subtype of RCC 20, 42, and it seemed to be more representative than BMI alone.

Despite the fact that computed tomography (CT) is used to be considered primarily standard imaging method for measurement of abdominal obesity, MRI is a highly predictive, accurate and safe modality to quantitatively assess body fat distribution 43-45, transcending the shortcomings of rough BMI cutoff. Therefore, as a routinely performed modality in ccRCC patients before surgery in our institution, MRI was utilized to measure SAT and then VAT% calculated.

Meanwhile, GSEA analysis illustrated that *CD36* associated with fatty acid metabolism, ultraviolet light response, angiogenesis and TGF-β signaling pathways were enriched in ccRCC tissues. Besides lipid metabolism, growing evidence has demonstrated that CD36, as an endogenous anti-inflammatory cytokine, helps to explain tumorigenesis through the immunomodulatory actions of ultraviolet light 46. Based on its participation in angiogenesis, one could speculate that *CD36* involved in invasion and metastasis of tumor cells 47, 48. CD36 may also contribute to the activation of latent TGF-β, to enhance inflammatory responses, and to suppress immune responses 49.

Strength of our study lay in our first attempt to investigate the role of *CD36* as a prognostic factor of ccRCC. Relationship between *CD36* and ccRCC was rarely documented, while it is noteworthy that *CD36* is estimated most expressed in many cancer patients. With this in mind, in the cohort of our study, we found dramatic *CD36* mRNA quantity contrast between 367 paired tumor and adjacent normal tissues, and first demonstrated that ccRCC patients with elevated *CD36* expression had shorter PFS and OS, which was similar to the results of some other tumors 50, 51. On the other hand, we first figure out that certain distribution of abdominal adipose depots in ccRCC patents, particularly VAT%, was positively correlated with over-expression of *CD36* mRNA. In addition, VAT% was access to predict progression and prognosis for ccRCC patients in this study, yet BMI failed. Therefore, it also provides a provoking thought that underlying adipose metabolism variation concerning VAT% might clarify the correlation triggering carcinogenesis. To further explain underlying ability of invasion and metastasis of *CD36*, data from public database were implemented with GSEA analysis to identify significant genes and pathways.

At the same time, the main limitations proposed in this study are obvious as follows. First, our research failed to deeply clarify the underlying mechanism of *CD36* involved in ccRCC. However, overall data indicate elevated *CD36* mRNA expression as one of progressive indicators and close association with poor prognosis of ccRCC. It also provides a plausible explanation that high *CD36* expression tied with advanced TNM stage and high ISUP grade, which were poor prognostic indicators of ccRCC. It may reflect to some extent that obesity-exposure causes cellular vital adipose metabolism variations and triggers carcinogenesis. While further investigations such as *CD36* gene methylation status and models of protein co-expression are required for mechanism analysis in the future research. Second, the abdominal adipose thickness at the level of the umbilicus was measured by MRI in this study, and then VAT% was directly calculated. This measurement method may have caused some calculation bias, with excess tissues including, bowel, bone, spinal fluid, muscle and visceral fat probably observed. Meanwhile, MRI scan was only available in 104 patients. Another limitation in this study is that generalizability and population variety may not conclusive enough to firmly support our results.

## Conclusion

In conclusion, our study first reveal that elevated *CD36* mRNA expression is positively correlated to distribution of abdominal adipose, particularly VAT%, which, in addition, notably predicts poor prognosis in ccRCC patients.

## Figures and Tables

**Table 1:** Clinicopathological characteristics in relation to *CD36* expression status.

Variable	Entire group (n=367)	*CD36* expression			*P* value
		Low expression (n=176)	Middle expression (n=124)	High expression (n=67)	
Age at surgery (y, mean±SD)	55.3±11.7	55.5±11.6	57.0±12.2	54.3±10.9	0.145
Sex (n, %)					0.860
Male	248 (67.6)	117 (66.5)	84 (67.7)	47 (70.1)	
Female	119 (32.4)	59 (33.5)	40 (32.3)	20 (29.9)	
BMI (kg/m^2^, mean±SD)	1.5±0.5	1.5±0.5	1.6±0.5	1.4±0.5	0.092
Clinical manifestation (n, %)					0.829
Incidental	246 (67.0)	118 (67.0)	85 (68.5)	43 (64.2)	
Symptomatic	121 (33.0)	58 (33.0)	39 (31.5)	24 (35.8)	
Laterality (n, %)					0.092
Left	182 (49.6)	92 (52.3)	52 (41.9)	38 (56.7)	
Right	185 (50.4)	84 (47.7)	72 (58.1)	29 (43.3)	
Tumor size (cm, mean±SD)	5.2±2.4	4.9±2.6	5.5±2.4	5.1±1.8	0.051
T stage at presentation (n, %)					0.003
T1-T2	300 (81.7)	150 (85.2)	105 (84.7)	45 (67.2)	
T3-T4	67 (18.3)	26 (14.8)	19 (15.3)	22 (32.8)	
N stage at presentation (n, %)					<0.001
N0	326 (88.8)	162 (92.0)	115 (92.7)	49 (73.1)	
N1	41 (11.2)	14 (8.0)	9 (7.3)	18 (26.9)	
M stage at presentation (n, %)					<0.001
M0	330 (89.9)	166 (94.3)	112 (90.3)	52 (77.6)	
M1	37 (10.1)	10 (5.7)	12 (9.7)	15 (22.4)	
ISUP grade (n, %)					0.023
1-2	175 (47.7)	92 (52.3)	61 (49.2)	22 (32.8)	
3-4	192 (52.3)	84 (47.7)	63 (50.8)	45 (67.2)	
Anthropometric measures of obesity on MRI (n=104, 28.3%)					
No. of Patients (%)*	104 (100)	59 (56.7)	32 (30.8)	13 (12.5)	
SAT (mm, mean±SD)	33.2±9.3	35.1±10.7	30.9±7.0	30.5±4.1	0.058
VAT (%, mean±SD)	83.6±3.2	82.8±3.5	84.5±2.5	85.4±1.9	0.004

* % was calculated according to the row, for other variables % was calculated according to the column. Abbreviations: BMI: body mass index; MRI: magnetic resonance imaging; SAT: subcutaneous adipose tissue; VAT: visceral adipose tissue.

**Table 2:** Univariate and multivariate Cox regression analyses of PFS in 367 enrolled ccRCC patients.

	Univariate analysis		Multivariate analysis	
Covariates	HR (95%CI)	P value	HR (95%CI)	P value
Age at surgery	**1.015 (1.003-1.027)**	**0.014**	1.006 (0.993-1.019)	0.336
Sex (male vs. female)	0.774 (0.567-1.058)	0.108	0.837 (0.605-1.160)	0.286
BMI (<25kg/m^2^ vs. ≥25kg/m^2^)	**1.371 (1.033-1.819)**	**0.029**	1.227 (0.911-1.652)	0.178
Clinical manifestation (incidental vs. symptomatic)	1.010 (0.751-1.359)	0.945	0.970 (0.711-1.324)	0.848
Laterality (left vs. right)	1.053 (0.795-1.393)	0.720	1.008 (0.755-1.345)	0.957
Tumor size	1.033 (0.979-1.090)	0.233	1.049 (0.987-1.115)	0.121
T stage (T1-T2 vs. T3-T4)	**10.296 (7.287-14.549)**	**<0.001**	**1.797 (1.282-2.519)**	**0.001**
N stage (N0 vs. N1)	**12.415 (8.356-18.444)**	**<0.001**	**3.340 (1.915-5.826)**	**<0.001**
M stage (M0 vs. M1)	**12.324 (8.227-18.461)**	**<0.001**	**4.510 (2.980-6.825)**	**<0.001**
ISUP grade (1-2 vs. 3-4)	**3.019 (2.225-4.098)**	**<0.001**	1.544 (0.867-2.750)	0.140
*CD36* expression (Low vs. middle)	**3.096 (2.195-4.366)**	**<0.001**	**2.656 (1.863-3.785)**	**<0.001**
*CD36* expression (low vs. high)	**4.873 (3.300-7.196)**	**<0.001**	**3.244 (2.152-4.890)**	**<0.001**

Abbreviations: PFS: progression free survival; ccRCC: clear cell renal cell carcinoma; HR: hazard ratio; CI: confidence interval; BMI: body mass index; ISUP: International Society of Urological Patheology.

**Table 3:** Univariate and multivariate Cox regression analyses of OS in 367 enrolled ccRCC patients.

	Univariate analysis			Multivariate analysis	
Covariates	HR (95%CI)	P value		HR (95%CI)	P value
Age at surgery	**1.018 (1.004-1.033)**	**0.012**		1.015 (0.999-1.030)	0.053
Sex (male vs. female)	0.922 (0.639-1.331)	0.665		1.028 (0.698-1.514)	0.889
BMI (<25kg/m^2^ vs. ≥25kg/m^2^)	1.255 (0.892-1.766)	0.192		1.082 (0.751-1.559)	0.673
Clinical manifestation (incidental vs. symptomatic)	0.968 (0.674-1.391)	0.861		0.943 (0.644-1.379)	0.761
Laterality (left vs. right)	1.030 (0.735-1.443)	0.866		0.873 (0.615-1.238)	0.446
Tumor size	0.995 (0.928-1.067)	0.890		1.025 (0.947-1.109)	0.542
T stage (T1-T2 vs. T3-T4)	**12.148 (8.359-17.654)**	**<0.001**		**1.572 (1.011-2.445)**	**0.045**
N stage (N0 vs. N1)	**12.684 (8.352-19.264)**	**<0.001**		**3.492 (1.944-6.273)**	**<0.001**
M stage (M0 vs. M1)	**11.554 (7.618-17.525)**	**<0.001**		**5.270 (3.256-8.532)**	**<0.001**
ISUP grade (1-2 vs. 3-4)	**3.466 (2.364-5.038)**	**<0.001**		1.384 (0.772-2.481)	0.276
*CD36* expression (Low vs. middle)	**2.113 (1.383-3.229)**	**0.001**		**1.925 (1.239-2.992)**	**0.004**
*CD36* expression (low vs. high)	**4.610 (2.956-7.189)**	**<0.001**		**2.491 (1.553-3.996)**	**<0.001**

Abbreviations: OS: overall survival; ccRCC: clear cell renal cell carcinoma; HR: hazard ratio; CI: confidence interval; BMI: body mass index; ISUP: International Society of Urological Pathology.
