# Supplementary material for: Elevated CD36 expression correlates with increased visceral adipose tissue and predicts poor prognosis in ccRCC patients
Source: J Cancer. 2019 Jul 25;10(19):4522–31. doi: 10.7150/jca.30989 (PMC6746135; doi:10.7150/jca.30989)
Supplement: Supplementary file 1 — Supplementary figures and tables. [file jcav10p4522s1.pdf]

Supplementary Figure 1. Flow chart summarized the criteria for inclusion and exclusion of a total of 405 ccRCC patients in this cohort.

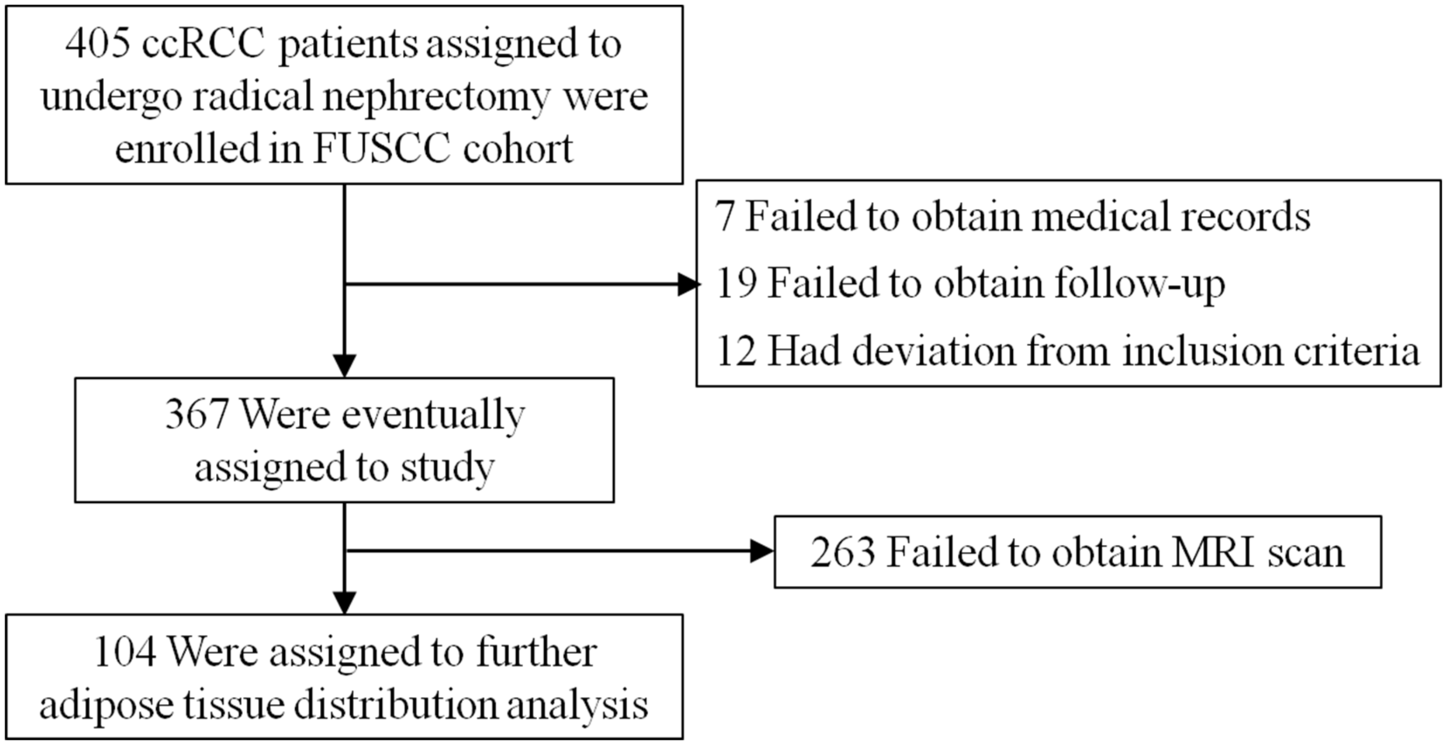

Supplementary Table 1. Univariate and multivariate Cox regression analyses of PFS in 104 cases whose MRI scans were available.

	Univariate analysis		Multivariate analysis	
Covariates	HR (95%CI)	P value	HR (95%CI)	P value
Age at surgery	1.015 (0.991-1.040)	0.230	1.023 (0.992-1.055)	0.146
Sex (male vs. female)	0.703 (0.378-1.308)	0.266	1.440 (0.643-3.223)	0.375
BMI (<25kg/m^2 vs. ≥25kg/m^2)	0.939 (0.516-1.706)	0.836	0.994 (0.952-1.038)	0.793
A	**0.907 (0.852-0.966)**	**0.003**	1.124 (0.818-1.543)	0.471
P	0.964 (0.911-1.021)	0.212	1.322 (0.956-1.827)	0.092
AP	0.998 (0.988-1.009)	0.796	0.975 (0.930-1.023)	0.298
SAT	**0.943 (0.904-0.984)**	**0.007**	1.184 (0.870-1.611)	0.283
VAT%	**3.427 (1.488-11.195)**	**<0.001**	**2.556 (1.036-6.310)**	**0.042**
Clinical manifestation (incidental vs. symptomatic)	0.949 (0.526-1.715)	0.864	0.772 (0.370-1.612)	0.491
Laterality (left vs. right)	0.906 (0.516-1.589)	0.731	1.008 (0.519-1.955)	0.981
Tumor size	**1.146 (1.034-1.270)**	**0.009**	0.899 (0.777-1.040)	0.151
T stage (T1-T2 vs. T3-T4)	**5.210 (2.307-11.766)**	**<0.001**	**2.164 (1.029-4.550)**	**0.042**
N stage (N0 vs. N1)	**6.700 (3.263-13.747)**	**<0.001**	**6.519 (1.867-22.759)**	**0.003**
M stage (M0 vs. M1)	**9.631 (4.964-18.687)**	**<0.001**	**20.911 (8.024-54.495)**	**<0.001**
ISUP grade (1-2 vs. 3-4)	1.777 (0.964-3.277)	0.065	0.288 (0.070-1.182)	0.084
CD36 expression (Low vs. middle)	**3.513 (1.849-6.672)**	**<0.001**	**2.578 (1.276-5.209)**	**0.008**
CD36 expression (low vs. high)	**4.544 (1.977-10.444)**	**<0.001**	**2.623 (1.088-6.324)**	**0.032**

Abbreviations: PFS, progression-free survival; MRI, magnetic resonance imaging; BMI, body mass index; ISUP, International Society of Urological Pathology.

Supplementary Table 2. Univariate and multivariate Cox regression analyses of OS in 104 cases whose MRI scans were available.

	Univariate analysis		Multivariate analysis	
Covariates	HR (95%CI)	P value	HR (95%CI)	P value
Age at surgery	1.024 (0.992-1.056)	0.139	1.044 (0.997-1.093)	0.066
Sex (male vs. female)	0.865 (0.407-1.836)	0.705	0.935 (0.428-2.040)	0.865
BMI (<25kg/m^2 vs. ≥25kg/m^2)	0.842 (0.396-1.790)	0.655	0.662 (0.267-1.641)	0.373
A	**0.921 (0.854-0.993)**	**0.032**	1.346 (0.866-2.092)	0.187
P	0.958 (0.887-1.035)	0.277	1.380 (0.908-2.096)	0.131
AP	0.998 (0.985-1.011)	0.744	0.942 (0.879-1.008)	0.086
SAT	**0.945 (0.898-0.995)**	**0.032**	**0.935 (0.875-0.999)**	**0.046**
VAT%	**3.150 (1.940-9.513)**	**0.005**	**3.291 (1.034-10.477)**	**0.044**
Clinical manifestation (incidental vs. symptomatic)	0.779 (0.359-1.694)	0.529	0.374 (0.126-1.106)	0.075
Laterality (left vs. right)	1.035 (0.511-2.098)	0.924	1.584 (0.639-3.928)	0.320
Tumor size	1.117 (0.985-1.266)	0.085	**0.761 (0.623-0.928)**	**0.007**
T stage (T1-T2 vs. T3-T4)	**6.961 (2.980-16.261)**	**<0.001**	2.526 (0.826-7.722)	0.104
N stage (N0 vs. N1)	**8.092 (3.656-17.908)**	**<0.001**	**28.315 (5.206-153.987)**	**<0.001**
M stage (M0 vs. M1)	**11.725 (5.532-24.850)**	**<0.001**	**39.736 (11.637-135.680)**	**<0.001**
ISUP grade (1-2 vs. 3-4)	1.876 (0.862-4.081)	0.113	0.646 (0.138-3.016)	0.578
CD36 expression (Low vs. middle)	**0.754 (1.204-6.300)**	**0.016**	2.325 (0.991-5.455)	0.053
CD36 expression (low vs. high)	**4.053 (1.508-10.895)**	**0.006**	**3.626 (1.332-9.868)**	**0.012**

Abbreviations: OS, overall survival; MRI, magnetic resonance imaging; BMI, body mass index; ISUP, International Society of Urological Pathology.
